# Reporting of Noninferiority Margins on ClinicalTrials.gov: A Systematic Review

**DOI:** 10.1001/jamanetworkopen.2025.3569

**Published:** 2025-04-07

**Authors:** Camille Reinaud, Sandra Mavoungou, David Hajage, Chloé Lieng, Deivanes Rajendrabose, Diane Ferreira, Jules Blanchard, Agathe Turpin, Agnès Dechartres

**Affiliations:** 1 Sorbonne Université, Institut National de la Santé et de la Recherche Médicale, Institut Pierre Louis d’Epidémiologie et de Santé Publique, Assistance Publique–Hôpitaux de Paris (AP-HP), Hôpital Pitié-Salpêtrière, Département de Santé Publique, Centre de Pharmacoépidémiologie de l’AP-HP (Cephepi), Paris, France

## Abstract

**Importance:**

The noninferiority margin is a key methodological parameter in noninferiority trials that affects both sample size calculation and interpretation of results.

**Objective:**

To assess (1) the reporting of the noninferiority margin on ClinicalTrials.gov, including when it was reported; (2) the consistency of the noninferiority margin between registration and publication; and (3) the reporting of the noninferiority margin at registration in a sample of recent trials.

**Evidence Review:**

This systematic review was conducted in 2 stages. Stage 1 involved identifying all noninferiority trials registered on ClinicalTrials.gov with a primary completion date of January 1, 2010, to January 1, 2015, and searching for corresponding publications. Stage 2 included all noninferiority trials registered between January 1, 2022, and June 30, 2023. Two reviewers independently and manually extracted methodological characteristics related to the noninferiority design for each trial at registration and, when available, in results posted on ClinicalTrials.gov and in publications. The time points for reporting the noninferiority margin were at registration, during the patient enrollment phase (between start date and primary completion date), after the primary completion date, or in results posted.

**Findings:**

Among the 266 trials completed between 2010 and 2015, only 8 (3.0%) reported the noninferiority margin at registration. The noninferiority margin was reported after registration for 31 of 266 trials (11.7%), with 11 (4.1%) reporting it during the patient enrollment phase and 20 (7.5%) reporting it after the primary completion date. Of the 132 trials with results posted on ClinicalTrials.gov, 79 (59.8%) reported the noninferiority margin. A corresponding publication was found for 208 trials, with 196 (94.2%) reporting the noninferiority margin. For 5 trials reporting the margin at both registration and in publication, the noninferiority margin was consistent in both sources. Among the 327 noninferiority trials first posted between 2022 and 2023, 30 (9.2%) reported the noninferiority margin at registration.

**Conclusions and Relevance:**

In this systematic review, the reporting of the noninferiority margin on ClinicalTrials.gov was low. Mandatory reporting of the design and the noninferiority margin at registration could enhance the transparency and favor more reliable results.

## Key Points

**Question:**

What proportion of noninferiority trials report the noninferiority margin at registration and in results posted on ClinicalTrials.gov compared with corresponding publications?

**Findings:**

In this systematic review of 266 trials completed between 2010 and 2015 and 327 trials first posted between 2022 and 2023, only 3.0% and 9.2% of trials, respectively, reported the noninferiority margin at registration on ClinicalTrials.gov. For the 2010 to 2015 sample, the proportions were higher for reporting in results posted on ClinicalTrials.gov and in corresponding publications.

**Meaning:**

The findings indicate that, despite its importance in trial planning and interpretation of results, the noninferiority margin is poorly reported on ClinicalTrials.gov.

## Teaser

This systematic review evaluates the proportion, time point, and consistency between registration and publication of noninferiority margin reporting.

## Introduction

Noninferiority trials aim to determine whether a new treatment is as effective as a reference treatment. The noninferiority margin is a central element in these trials, representing the loss of effectiveness in the primary end point compared with the reference treatment, which is acceptable given the benefits offered by the new treatment, such as improved safety, lower costs, or more convenient administration schemes. The importance of this margin and the methods for its definition have been described in guidelines from the US Food and Drug Administration,^1^ the European Medicines Agency,^2^ and the CONSORT (Consolidated Standards of Reporting Trials) 2010 statement extension for noninferiority trials.^3^ The margin must be justified on the basis of existing literature or clinical relevance and established during the trial planning stage for sample size calculation. The noninferiority margin is also crucial for interpreting results and drawing conclusions. To establish the noninferiority of the new treatment compared with the reference treatment, the CI of the new treatment effect must lie within the zone defined by the noninferiority margin.

Since 2005, the International Committee of Medical Journal Editors has mandated the registration of clinical trials in recognized public registries as a prerequisite for publication.^4,5^ One of the objectives of the prospective registration of clinical trials is to limit selective outcome reporting, a practice that involves modifying outcomes between the protocol and the publication, which has been shown to distort research findings toward overly favorable and misleading results.^6,7,8,9,10^ Given the crucial role of the noninferiority margin in the design and interpretation of noninferiority trials, its reporting at the time of registration as well as its consistency through the publication appears essential. However, concerns have been raised regarding the potential distortion of the noninferiority margin in protocols, trial registries, and publications. A study published in 2015 identified a discordance rate of 9% between the noninferiority margins reported in protocols submitted to ethics committees and those reported in corresponding publications.^11^ Few data exist on the reporting of the noninferiority margin in trial registries, but the most recent study found that a low proportion (only 2.6%) of the trials prespecified the noninferiority margin at initial registration.^12^ These findings were based on trials published between 2012 and 2014; thus, their registration on ClinicalTrials.gov occurred much earlier.

To our knowledge, no study has addressed this issue within a more recent time frame. In this study, we aimed to assess (1) the reporting of the noninferiority margin on ClinicalTrials.gov, including when it was reported; (2) the consistency of the noninferiority margin between registration and publication; and (3) the reporting of the noninferiority margin at registration in a sample of recent trials.

## Methods

This systematic review was conducted in 2 stages in accordance with the Preferred Reporting Items for Systematic Reviews and Meta-Analyses (PRISMA) reporting guideline.^13^ First, studies were selected based on a primary completion date (ie, the date of the final data collection for the primary outcome of the last included patient) of January 1, 2010, to January 1, 2015, to allow sufficient time for publication. For these studies (hereafter the 2010 to 2015 sample), the reporting of the noninferiority margin was assessed along with the timing of its first mention: at registration, during the patient enrollment phase, after the primary completion date, or in results posted. Corresponding publications were then searched to ascertain whether the noninferiority margin was reported and whether it was consistent with that reported on ClinicalTrials.gov. Second, the reporting of the noninferiority margin at registration was examined in a recent sample of trials with a first posted date (ie, the date on which the study record was first available on ClinicalTrials.gov) of January 1, 2022, to June 30, 2023, to assess any improvement in the reporting of the noninferiority margin at registration. These studies are referred to as the 2022 to 2023 sample.

### Registered Trials With a Primary Completion Date of January 1, 2010, to January 1, 2015

#### Stage 1: Identification of Noninferiority Trials on ClinicalTrials.gov

ClinicalTrials.gov was searched for noninferiority trials with a primary completion date of January 1, 2010, to January 1, 2015, using the keyword *non-inferiority*, specifying *interventional study* as the study type and *completed* as the recruitment status. Inclusion criteria were noninferiority randomized clinical trials assessing therapeutic interventions, thereby excluding all interventions related to diagnosis or screening. Nonrandomized trials, single-arm studies, and phase 1, 1-2, 2, or 2-3 trials were excluded to ensure a more homogeneous sample focusing on noninferiority trials in phase 3 or 4, which are the most common setting for conducting such studies. Each record was reviewed to verify eligibility. Trial reports in which noninferiority was mentioned as a secondary outcome were excluded. Additionally, duplicates of trials that were registered more than once were removed.

#### Stage 1: Data Extraction on ClinicalTrials.gov

For all included noninferiority trials, the extracted characteristics from ClinicalTrials.gov included general characteristics, such as trial registration number (National Clinical Trial [NCT] number), first posted date, start date, primary completion date, and primary funding source. Trial design details were also collected, including study phase (phase 3, phase 4, or not applicable for trials not evaluating a drug), number of arms, study design (parallel arms, crossover, or factorial), masking, and sample size. Clinical characteristics were collected, such as age, sex, and medical condition. Additionally, information on treatments evaluated in the experimental and control groups as well as the primary outcome were extracted.

Two reviewers (S.M. and C.L.) independently and manually extracted methodological characteristics for each trial from ClinicalTrials.gov. The extraction also identified whether the noninferiority margin was reported in any fields and whether it was justified with a reference publication. If a noninferiority margin was reported, the history of changes was examined to identify at which stage the margin was reported for the first time on ClinicalTrials.gov: at registration, during the patient enrollment phase (between start date and primary completion date), or after the primary completion date. The primary analysis population, distinguished as per protocol (PP), intention to treat (ITT), or modified intention to treat (mITT), was also extracted. The type I error rate reported in sample size calculation was also extracted. Additionally, the presence of posted results—and if available, whether the noninferiority margin was reported in the results—was assessed. Information on the analysis population and type I error reported in study results was also collected.

#### Stage 1: Search for Corresponding Publications

For each trial, 2 reviewers (including S.M., A.T., or C.R. and C.L., D.R., D.F., or J.B.) independently and manually searched for corresponding publications published until November 15, 2023, using the link provided on ClinicalTrials.gov. If no link was reported, Google Scholar was searched using the NCT number. Subsequently, MEDLINE was searched via PubMed using keywords related to the experimental treatment and the condition under study. To ascertain whether an article partially or completely matched the registered trial data, a combination of information was verified, such as trial acronym, description of interventions and population, location, responsible party, number of participants, trial phase, primary outcome measures, and primary funding sponsor. In case of doubt, a senior researcher (A.D.) was consulted to solve disagreements and achieve consensus.

#### Stage 1: Data Extraction in Corresponding Publications

Two reviewers (including S.M., A.T., or C.R. and C.L., D.R., D.F., or J.B.) independently collected data from all elements of the publication, including online supplements. The extracted information included general characteristics of the publication, such as the journal name, first author, first date of online publication, and type of journal (general or specialty). Details on treatments evaluated in the experimental and control groups, number of arms, number of patients randomized and analyzed, and definition of the primary outcome were collected. Methodological characteristics related to the noninferiority design were examined, including the noninferiority margin, whether it was justified or not, and how it was justified. The primary analysis population was assessed to determine whether it was based on an ITT, a PP, or an mITT analysis. Additionally, the type I error rate was extracted.

### Registered Trials With a First Posted Date of January 1, 2022, to June 30, 2023

#### Stage 2: Identification of Noninferiority Trials on ClinicalTrials.gov

A recent sample of noninferiority trials, with a first posted date of January 1, 2022, to June 30, 2023, was selected from ClinicalTrials.gov using the keyword *non-inferiority*, specifying *interventional study* as the study type and *all* as study status. The same inclusion criteria as the 2010 to 2015 sample were applied.

#### Stage 2: Data Extraction on Clinical Trials.gov

The data extracted from ClinicalTrials.gov for the 2022 to 2023 sample were the same as those for the 2010 to 2015 sample: general characteristics, trial design, clinical characteristics, and data on treatments evaluated. Two reviewers (C.R. and D.F. or J.B.) independently and manually extracted methodological characteristics for each trial. These characteristics included whether the noninferiority margin was reported at initial registration in any fields and whether it was justified with a reference publication, the primary analysis population, and the type I error rate as reported in the sample size calculation.

### Statistical Analysis

Descriptive analysis involved calculating frequencies and percentages for qualitative variables and medians (IQRs) for quantitative variables. Trials registered with a noninferiority design that were reported as superiority or equivalence trials in the publication were included in the registration and results-posted analysis but were excluded from the publication analysis.

We used the Kaplan-Meier method to assess time (in months) between the primary trial completion date and the online publication date. Trials for which the results were not published by November 15, 2023, were censored on this date. We used R, version 4.3.3 (R Project for Statistical Computing) for statistical analysis.

## Results

### 2010 to 2015 Sample of Registered Trials 

**Figure 1.**
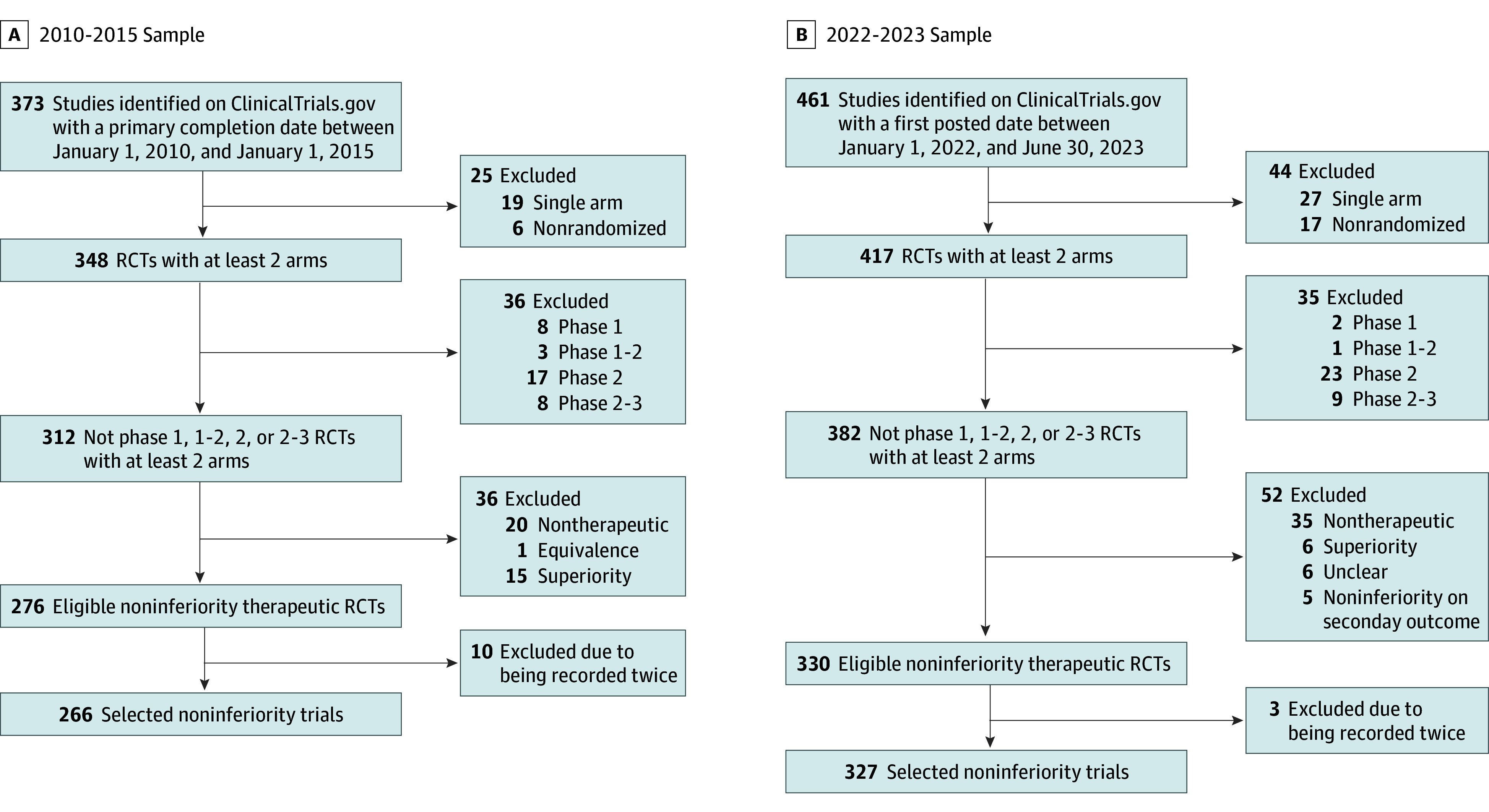
Flowchart of the Selection of Trials RCT indicates randomized clinical trial.

The stage 1 search identified 373 studies registered on ClinicalTrials.gov. After excluding 107 studies that did not meet the inclusion criteria, the final 2010 to 2015 sample consisted of 266 trials (Figure 1 and eTable 1 in Supplement 1). The primary funding source was industry for 160 trials (60.2%) (Table 1). Two hundred six trials (77.4%) evaluated drugs as the experimental treatment. Regarding design, 143 trials (53.8%) were phase 3, 250 (94.0%) had parallel arms, and 129 (48.5%) were open label. Two hundred seventeen trials (81.6%) had 2 arms, and only 49 (18.4%) had 3 or more arms; among these 49 trials, 6 (12.2%) had a control group with no active treatment. The median (IQR) planned sample size was 304 (63-545), and the median (IQR) time between the trial start date and primary completion date was 17.0 (6.0-28.0) months.

**Table 1.** Characteristics of Included Trials on ClinicalTrials.gov

Characteristic	Registered trials, No. (%)	
	Primary completion date between 2010 and 2015 (n = 266)	First posted date between 2022 and 2023 (n = 327)
Primary funding source		
Industry	160 (60.2)	85 (26.0)
Academic	83 (31.2)	213 (65.1)
Academic and industry	20 (7.5)	2 (0.6)
Other: NIH	3 (1.1)	27 (8.3)
Type of experimental intervention		
Drugs, including biological	206 (77.4)	131 (40.1)
Devices	33 (12.4)	74 (22.6)
Procedures	9 (3.4)	57 (17.5)
Behavioral, including diet	5 (1.9)	22 (6.7)
Other^a^	13 (4.9)	43 (13.1)
Study phase		
Phase 3	143 (53.8)	80 (24.5)
Phase 4	61 (23.0)	52 (15.9)
NA	62 (23.3)	195 (59.6)
Study design		
Parallel arms	250 (94.0)	316 (96.6)
Crossover	13 (4.9)	9 (2.8)
Factorial	3 (1.1)	2 (0.6)
No. of arms		
2	217 (81.6)	279 (85.3)
≥3	49 (18.4)	48 (14.7)
Control group with no active treatment		
Yes	6 (2.3)	8 (2.4)
No	260 (97.7)	319 (97.6)
Masking		
Open label	129 (48.5)	161 (49.2)
Single	30 (11.3)	73 (22.3)
Double	27 (10.1)	47 (14.4)
≥Triple	80 (30.1)	46 (14.1)
Population included		
Adults only	196 (73.7)	271 (82.8)
Children only	44 (16.5)	28 (8.6)
All	26 (9.8)	28 (8.6)
Sex		
Male and female	244 (91.7)	288 (88.1)
Female	20 (7.5)	33 (10.1)
Male	2 (0.8)	6 (1.8)
Planned sample size, median (IQR)	304 (63-545)	228 (50-406)
Time between start date and primary completion date, median (IQR), mo	17.0 (6.0-28.0)	NA

Abbreviations: NA, not applicable; NIH, National Institutes of Health.

^a^ Other is an existing category on ClinicalTrials.gov.

**Figure 2.**
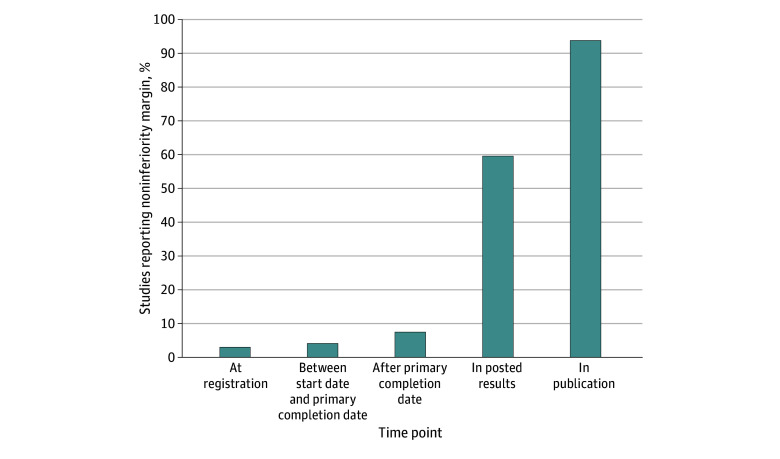
Reporting of the Noninferiority Margin at Different Time Points on ClinicalTrials.gov and in Corresponding Publications

Of the 266 included studies, only 8 (3.0%) reported the planned noninferiority margin at registration. For 31 studies (11.7%), initial reporting of the margin occurred after registration: 11 (4.1%) reported between the study start date and the primary completion date, and 20 (7.5%) reported after the primary completion date (Figure 2). None of these studies provided a justification for the choice of the noninferiority margin. In 227 studies (85.3%), the noninferiority margin was not reported in the study record.

Only 13 trials (4.9%) reported the planned primary analysis population at registration: 8 specified a PP, 2 specified an ITT, and 3 specified an mITT primary analysis. Only 10 trials (3.8%) reported the planned type I error. Of the 132 trials with results posted on ClinicalTrials.gov, 79 (59.8%) reported the noninferiority margin in their results (Table 2).

**Table 2.** Noninferiority-Related Methodological Characteristics on ClinicalTrials.gov and in Publications for the 2010 to 2015 Sample

Characteristic	2010-2015 Sample of trials, No. (%)		
	At registration (n = 266)	In results posted on ClinicalTrials.gov (n = 132)	In publications (n = 208)
Noninferiority margin reported	8 (3.0)	79 (59.8)	196 (94.2)
Justification of the margin	0	NA	86 (41.3)
With references	0	NA	80 (93.0)
With clinical experience	0	NA	6 (7.0)
Primary analysis population	13 (4.9)	103 (78.0)	195 (93.7)
PP	8 (61.5)	54 (52.4)	108 (55.4)
ITT	2 (15.4)	24 (23.3)	70 (35.9)
mITT	3 (23.1)	25 (24.3)	17 (8.7)
Type I error reported	10 (3.8)	32 (24.2)	145 (69.7)
2.5% 1-Sided	2 (20.0)	12 (37.5)	51 (35.2)
5.0% 1-Sided	3 (30.0)	3 (9.4)	32 (22.1)
2.5% 2-Sided	0	1 (3.1)	4 (2.7)
5.0% 2-Sided	2 (20.0)	8 (25.0)	24 (16.6)
2.5% Unspecified	0	3 (9.4)	7 (4.8)
5.0% Unspecified	3 (30.0)	5 (15.6)	24 (16.6)
Other^a^	0	0	3 (2.0)

Abbreviations: ITT, intention to treat; mITT, modified intention to treat; NA, not applicable; PP, per protocol.

^a^ Other reported type I error included 1.7% unspecified, 1.25% 1-sided, and unspecified.

A publication was identified for 212 of 266 studies (79.7%). Of these 212 studies, 4 (1.9%) were not reported as noninferiority trials in the publication: 3 were reported as superiority trials and 1 was reported as an equivalence trial. The median time from the primary trial completion date to publication was 35 (95% CI, 30-41) months (eFigure 1 in Supplement 1).

Among the 208 corresponding published noninferiority trials, the noninferiority margin was reported in 196 (94.2%) and was justified in 86 (41.3%), 80 (93.0%) of which cited previous studies (Table 2; eFigure 2 in Supplement 1). The primary analysis population was reported in 195 trials (93.7%): 108 reported a PP, 70 reported an ITT, and 17 reported an mITT. Among the 103 trials reporting the primary analysis in results posted, the analysis was consistent in the final publication for 66 trials (64.1%) (eFigure 3 in Supplement 1). Only 145 publications (69.7%) reported the type I error, with 51 (35.2%) reporting a 2.5% 1-sided error and 32 (22.1%) reporting a 5% 1-sided error (Table 2).

For the 5 trials that reported the noninferiority margin at registration and in the corresponding publication, the margin was identical in both sources. For all but 1 of the 63 trials that reported the noninferiority margin in the results posted on ClinicalTrials.gov and in the corresponding publication, the margin was consistent in both sources.

### 2022 to 2023 Sample of Registered Trials 

The stage 2 search identified 461 studies registered on ClinicalTrials.gov. After excluding 134 studies, the final 2022 to 2023 sample comprised 327 trials (Figure 1 and eTable 2 in Supplement 1). The primary funding source was academic for 213 trials (65.1%). One hundred thirty-one of 327 trials (40.1%) evaluated drugs as the experimental intervention. In terms of design, 316 (96.6%) had parallel arms and 161 (49.2%) were open label. Two hundred seventy-nine trials (85.3%) had 2 arms, and only 48 (14.7%) had 3 or more arms, of which 8 had a control group with no active treatment. The median (IQR) planned sample size was 228 (50-406) (Table 1).

The planned noninferiority margin was reported at registration in 30 of 327 trials (9.2%), 6 of which justified the margin. Only 11 trials (3.4%) reported the planned primary analysis population: 3 reported a PP, 4 reported an ITT, 1 reported an mITT, and 3 reported both a PP and an ITT without further precision. Only 13 trials (4.0%) reported the planned type I error.

## Discussion

To our knowledge, this systematic review was the first to address the reporting of the noninferiority margin from initial registration to publication. In the 2010 to 2015 sample of trials, a detailed longitudinal analysis from registration to publication found that the noninferiority margin was reported at registration on ClinicalTrials.gov in only 3.0% and after registration in 11.7% of studies. In addition, the noninferiority margin was underreported in results posted on ClinicalTrials.gov (59.8%), making the interpretation of results challenging. In contrast, the noninferiority margin was better reported in publications (94.2%). In the 2022 to 2023 sample, current reporting practices were assessed by examining a set of recently registered noninferiority trials. A slight but insufficient improvement was observed, with only 9.2% of trials reporting the noninferiority margin at registration.

The validity of these findings is reinforced by the methodological rigor we used: 2 authors independently selected trials, extracted data, and searched for corresponding publications, with discrepancies resolved by consensus with a third author. We explored the history of changes to identify the stage at which the margin was reported, which allowed us to explain how authors use ClinicalTrials.gov to declare the noninferiority margin and report results. The use of 2 distinct samples—1 allowing an assessment of the reporting of the margin until publication and 1 considering recently registered trials—allowed us to provide evidence of any improvement over time.

Previous studies have addressed the issue of the reporting of the noninferiority margin in trial registries. Dekkers et al^11^ analyzed registry records of noninferiority trials published between 2005 and 2009 and found that only 2.0% reported the noninferiority margin in trial registries. Gopal et al,^12^ in a cross-sectional analysis of noninferiority trials published between 2012 and 2014 and search for corresponding ClinicalTrials.gov records, found a consistent rate of noninferiority margin reporting of 2.6% at registration. The present study shows a slight improvement (albeit insufficient), with only 9.2% of studies reporting the noninferiority margin at registration in the 2022 to 2023 sample, and adds important information about the time at which the margin is reported.

This study highlights a persistent low rate of noninferiority margin reporting at registration on ClinicalTrials.gov. This absence of transparent and traceable reporting creates the possibility of modifications to the noninferiority margin after the trial has begun. Such changes are particularly concerning because they could skew results toward more favorable conclusions. The conclusion of a noninferiority trial—whether the results fall within or beyond the noninferiority margin—depends on the predefined margin value. Modifying this noninferiority margin post hoc, whether intentionally or not, allows treatments that initially failed to meet the noninferiority criteria to be later considered noninferior with an expanded margin. Altering the noninferiority margin during a trial, particularly after seeing the results, may be similar to selective outcome reporting. We did not search for the noninferiority margin in study protocols, which might have shown a higher reporting rate, but study protocols are not always available. ClinicalTrials.gov remains a more accessible source of trial information.

Consequently, we recommend making the reporting of the noninferiority margin mandatory on registration; otherwise, the margin will likely not be reported. To this end, we suggest that ClinicalTrials.gov and other registries introduce mandatory fields for the design of interventional trials, prompting authors to classify them as superiority, noninferiority, or equivalence trials. For noninferiority trials, mandatory fields related to the noninferiority margin may be implemented to facilitate the tracking of any changes to the noninferiority margin over time. Such a requirement would enhance not only the integrity of clinical research but also the trust in noninferiority trial results. This suggestion extends to equivalence trials.

### Limitations

This study has several limitations. First, we focused on ClinicalTrials.gov; therefore, the results may not be generalizable to other trial registries. Second, ClinicalTrials.gov does not currently provide a standardized design category to identify noninferiority trials; as a result, our search could have missed some of these trials. Third, despite careful data extraction in duplicate, we cannot exclude that we missed some noninferiority margins because they could be reported in any fields of ClinicalTrials.gov. Fourth, we did not search for noninferiority-related information in study protocols. Fifth, the 2 samples were not entirely comparable. In the 2022 to 2023 sample, many studies had not yet reached their primary completion date; thus, we were unable to describe the precise timing of noninferiority margin reporting on ClinicalTrials.gov and focused only on reporting at registration.

## Conclusions

In this systematic review, we identified a concerning issue regarding the poor reporting of noninferiority margins at registration on ClinicalTrials.gov. This lack of transparency may allow for untraceable changes to the noninferiority margins, potentially distorting conclusions toward more favorable results. Implementing mandatory reporting of the design and the noninferiority margin (for noninferiority trials and the equivalence margin for equivalence trials) at registration could enhance the transparency and favor more reliable results.

## Notes

Supplement 1. **eFigure 1.** Cumulative Incidence of Publication of Completed Non-inferiority Trials Registered on ClinicalTrials.gov Since Primary Trial Completion Date, for Studies With a Primary Completion Date From January 1, 2010 to January 1, 2015 **eFigure 2.** Reporting of the Non-inferiority Margin From Registration to Publication **eFigure 3.** Consistency of the Primary Analysis Population Between Results Posted and Corresponding Publications **eTable 1.** List of the ClinicalTrials.gov Records for the 2010 to 2015 Sample **eTable 2.** List of the ClinicalTrials.gov Records for the 2022 to 2023 Sample

Supplement 2.
Data Sharing Statement
