# Supplementary material for: Reporting of Noninferiority Margins on ClinicalTrials.gov: A Systematic Review
Source: JAMA Netw Open. 2025 Apr 7;8(4):e253569. doi: 10.1001/jamanetworkopen.2025.3569 (PMC11976490; doi:10.1001/jamanetworkopen.2025.3569)
Supplement: Supplement 1. — eFigure 1. Cumulative Incidence of Publication of Completed Non-inferiority Trials Registered on ClinicalTrials.gov Since Primary Trial Completion Date, for Studies With a Primary Completion Date From January 1, 2010 to January 1, 2015 eFigure 2. Reporting of the Non-inferiority Margin From Registration to Publication eFigure 3. Consistency of the Primary Analysis Population Between Results Posted and Corresponding Publications eTable 1. List of the ClinicalTrials.gov Records for the 2010 to 2015 Sample eTable 2. List of the ClinicalTrials.gov Records for the 2022 to 2023 Sample [file jamanetwopen-e253569-s001.pdf]

Supplemental Online Content

Reinaud C, Mavoungou S, Hajage D, et al. Reporting of noninferiority margins on ClinicalTrials.gov: a systematic review. *JAMA Netw Open*. 2025;8(4):e253569. doi:10.1001/jamanetworkopen.2025.3569

eFigure 1. Cumulative Incidence of Publication of Completed Non-inferiority Trials Registered on ClinicalTrials.gov Since Primary Trial Completion Date, for Studies With a Primary Completion Date From January 1, 2010 to January 1, 2015

eFigure 2. Reporting of the Non-inferiority Margin From Registration to Publication

eFigure 3. Consistency of the Primary Analysis Population Between Results Posted and Corresponding Publications

eTable1. List of the ClinicalTrials.gov Records for the 2010 to 2015 Sample

eTable 2. List of the ClinicalTrials.gov Records for the 2022 to 2023 Sample

This supplemental material has been provided by the authors to give readers additional information about their work.

© 2025 Reinaud C et al. *JAMA Network Open.*

eFigure 1. Cumulative incidence of publication of completed non-inferiority trials registered on ClinicalTrials.gov since primary trial completion date, for studies with a primary completion date from January 1, 2010 to January 1, 2015

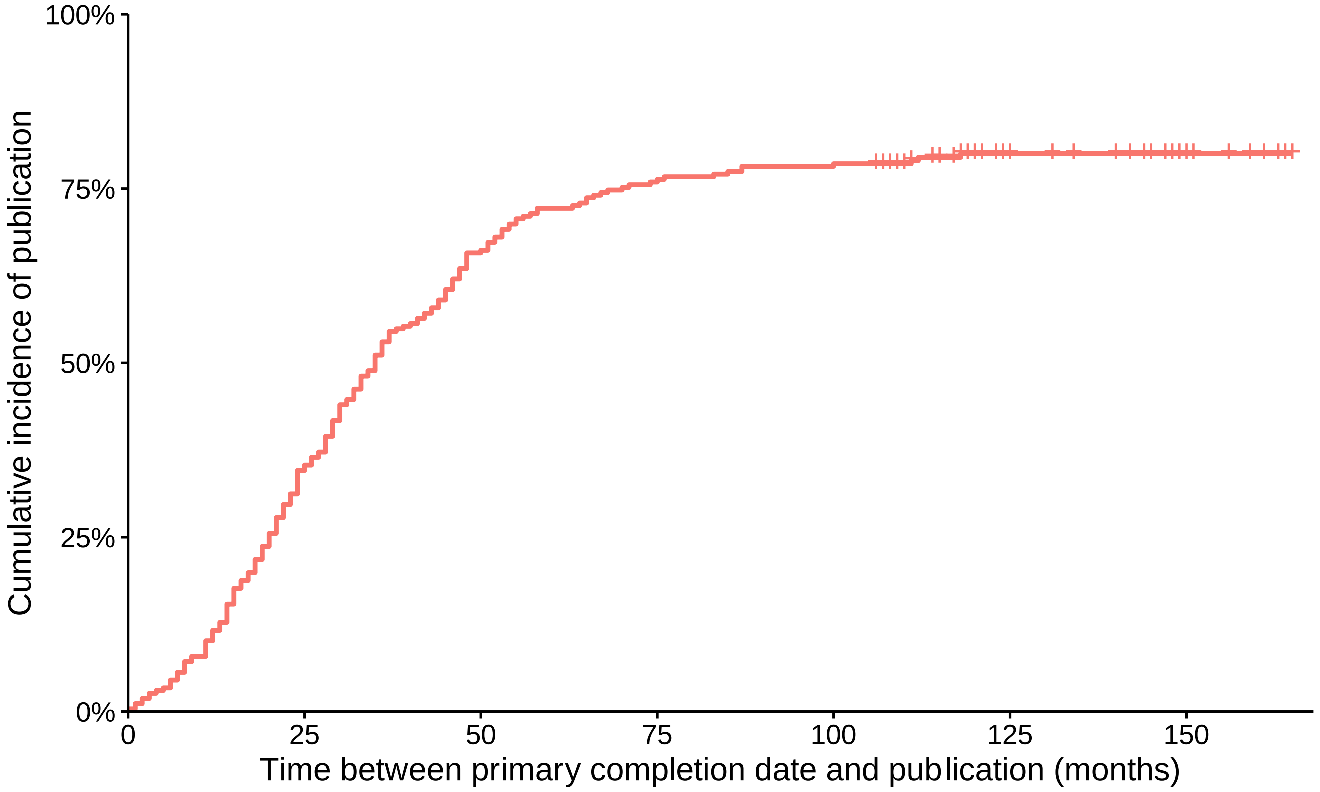

Number of articles still to be published

	266	172	90	64	58	27	10

© 2025 Reinaud C et al. *JAMA Network Open.*

eFigure 2. Reporting of the non-inferiority margin from registration to publication

Each spoke represents one of the trials registered on ClinicalTrials.gov with a primary completion date from January 1, 2010 to January 1, 2015. The 4 trials registered as NI trials but published as superiority or equivalence trials are not presented here. Every concentric circle represents one stage, with “at registration” being the central circle and “in publication” the furthest circle from the center.

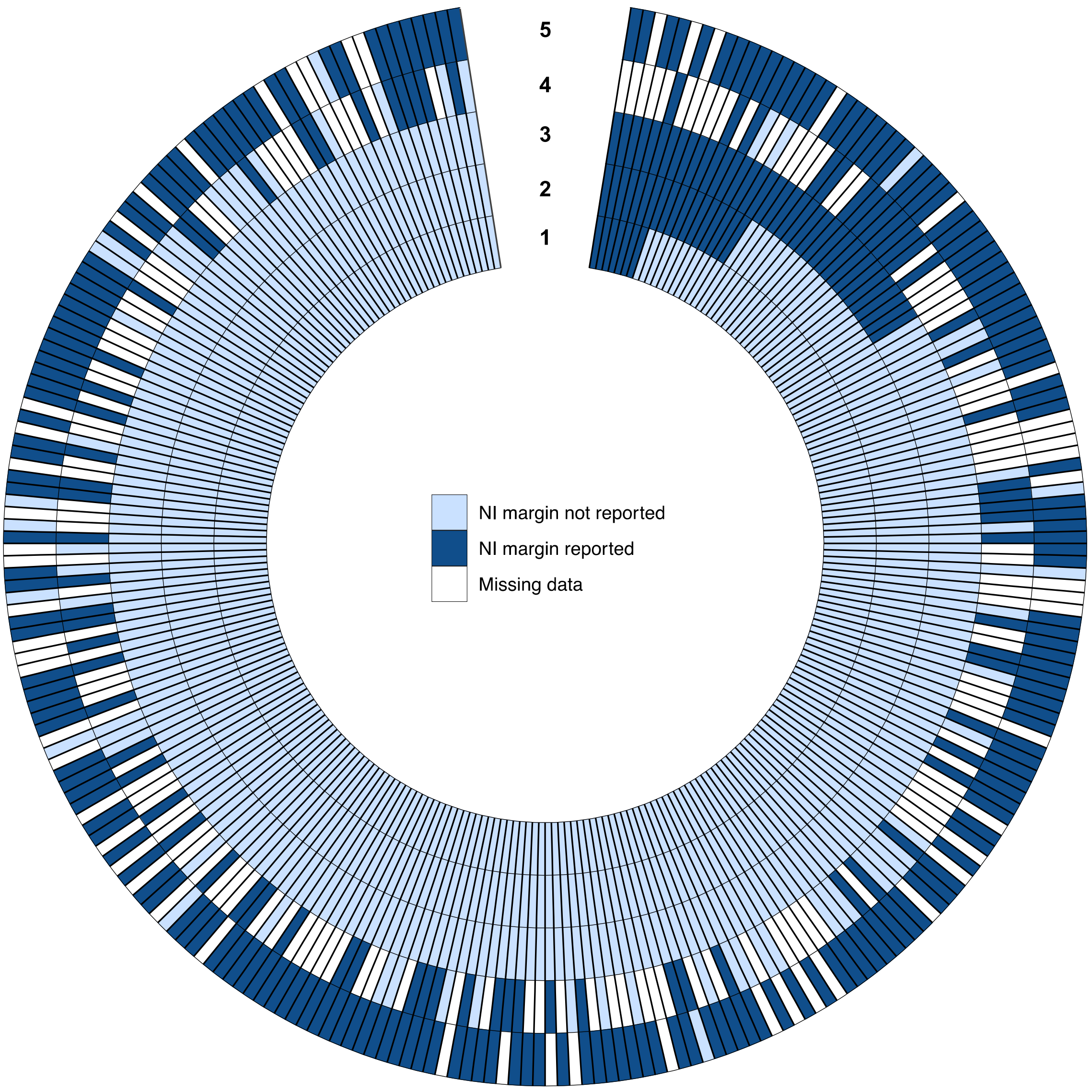

1: At registration

2: Between the study start date and the primary completion date

3: After the primary completion date

4: In results posted

5: In corresponding publication

© 2025 Reinaud C et al. *JAMA Network Open.*

eFigure 3. Consistency of the primary analysis population between results posted and corresponding publications

PP: per protocol; ITT: intention to treat; mITT: modified intention to treat

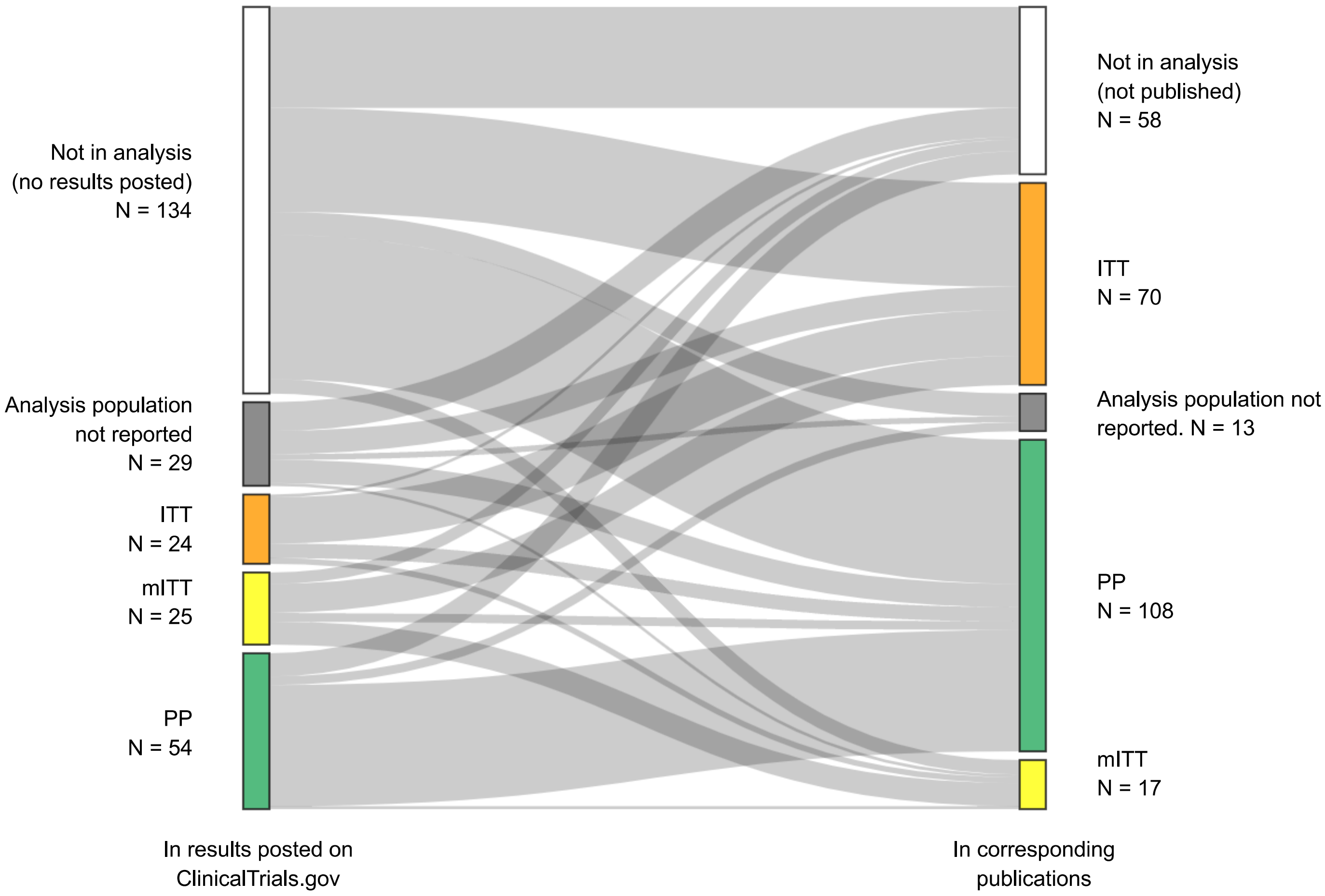

© 2025 Reinaud C et al. *JAMA Network Open.*

eTable1. List of the ClinicalTrials.gov records for the 2010 to 2015 sample

NCT	Published	Date of online publication	Journal	First author
NCT01180348	0			
NCT02670772	1	01/02/2019	Journal of Acquired Immune Deficiency Syndromes	Venter DWF
NCT01127100	1	04/02/2019	Pain research and management	Hwang CJ
NCT01576419	1	16/02/2014	Rheumatology international	Yoo WH
NCT00530738	1	30/11/2018	Nutrition Journal	Bohnert H
NCT00143390	1	30/05/2013	Breast cancer research and treatment	Iwata h
NCT00858637	1	15/09/2014	Blinical nephrology	Wanner c
NCT01399125	1	25/03/2016	cns neurosciences and therapeutics	Zhang zx
NCT01312766	1	20/04/2017	Reproductive biomedicine online	Lockwood G
NCT01449422	1	26/03/2014	Journal of wound care	Meaume s
NCT01188343	1	12/03/2014	Vaccine	Huang lm
NCT01881568	1	03/12/2014	The journal of bone and joint surgery	Gomez-Barrena E
NCT01257074	0			
NCT01931748	1	28/07/2023	Medicina	Seongmin Kim
NCT01083485	1	01/10/2012	The Journal of International Medical Research	Kuusniemi K
NCT01079676	1	27/07/2016	CLINICS	Roberto HEGG
NCT00902837	0			
NCT01599806	1	16/06/2016	Clinical Infectious disease	Wagenlehner FM
NCT01167010	0			
NCT01022047	0			
NCT01145339	0			
NCT01070043	1	17/07/2020	Journal of the Chinese Medical Association	Kang-Ling Wang
NCT01595438	1	16/06/2016	Clinical Infectious disease	Wagenlehner FM
NCT02016716	0			
NCT01511809	1	05/03/2016	Journal of antimicrobial chemotherapy	Galli L
NCT01841697	1	17/01/2017	Diabetes. obesity and metabolism	Goldenberg R
NCT01227824	1	08/01/2013	The lancet	Raffi F
NCT01464424	1	28/11/2014	BMC Ophtalmology	Dubiner HB
NCT00781391	1	19/11/2013	NEJM	Guigliano RP
NCT01145586	1	15/10/2016	Arq Gastroenterol	FRAESCONI F

© 2025 Reinaud C et al. *JAMA Network Open.*

NCT01307488	1	08/06/2015	Lancet Infectious disease	Perez-Molina
NCT01100164	1	15/01/2016	Clinics (Sao Paulo)	Pinto LR Jr
NCT01025817	1	04/03/2017	American Journal of Transplantation	F.Shihab
NCT01887405	0			
NCT02101476	0			
NCT01817556	0			
NCT00950664	1	05/12/2014	Movement Disorders	J.Young Yun
NCT01175902	1	12/01/2016	PLOS One	Na Young Lee
NCT02726269	1	27/07/2018	Revista de gastroenterologia de Mexico	L.Ladron-de-guevara
NCT02741024	1	06/09/2016	Malaria journal	M.de Wit
NCT01462422	1	01/09/2015	BJOG An International Journal of Obstetrics and Gynaecology	S Raghavan
NCT01231113	1	28/05/2017	Tropical Medicine in Health	J Osarfo
NCT00403767	1	10/08/2011	NEJM	
NCT00823836	1	01/10/2014	Neurology and Clinical Neuroscience	Nobutaka Hattori
NCT01709903	1	05/06/2015	International Journal of COPD	Nanshan Zhong
NCT01306461	1	16/04/2014	Journal of ocular pharmacology and therapeutics	G.Hollo
NCT01099722	1	26/10/2012	Journal of Asthma	ANNA BODZENTA-LUKASZYK.
NCT01159223	1	24/05/2016	Lancet HIV	Torsak Bunupuradah
NCT01923831	1	20/09/2015	International Journal of medical sciences	Jin Ha Park
NCT01804361	0			
NCT02024867	1	11/11/2015	The journal of pediatrics	Lucy Holmes.
NCT01425853	1	14/01/2015	Annals Rheumatology Disease	Marc C Hochberg
NCT01848054	1	07/06/2016	Journal of Addictive Diseases	L.Webster
NCT01540136	1	28/02/2018	Lancet Oncology	Lin-Quan Tang
NCT01399723	1	30/12/2014	Clinical Infectious disease	A. Agweyu
NCT01713426	1	01/12/2017	European journal of pain	M. Haanpaa
NCT00541658	1	27/09/2011	Osteoporosis international	McClung MR
NCT01058174	1	17/12/2014	Clinical Infectious disease	Saliba F
NCT01001364	0			
NCT01372865	1	15/02/2016	Brazilian journal of othorhinolaryngology	Antila MA
NCT01836224	1	16/09/2016	Liver international	Choudhury A

© 2025 Reinaud C et al. *JAMA Network Open.*

NCT01565265	0			
NCT01202084	1	15/11/2014	Journal brasilieiro de pneumologia	Antilla M
NCT00442702	0			
NCT00928187	1	15/07/2015	Aids	Ciaffi L
NCT01908842	1	26/09/2015	Clinical therapeutics	Gunderson E.W
NCT01912807	1	21/07/2020	Canadian Journal of Anaethesia	Vasquez-Camargo A
NCT00603733	0			
NCT00979628	1	22/02/2013	Diabetes care	Umpierrez G.E.
NCT01914926	1	22/04/2015	Pharmacology in emergency medicine	
NCT00818610	1	06/10/2014	JAMA internal medicine	Garin N
NCT01075282	1	18/06/2015	Diabetes care	Giorgino F
NCT00640562	1	01/05/2014	International clinical psychopharmacology	Di Fiorino M
NCT01574651	1	12/02/2015	BMJ	Roland BUHL
NCT00300274	1	22/02/2013	American Journal of Transplantation	Elsen HJ
NCT01495013	1	20/10/2015	Diabetic Medicine	Ambery P
NCT01867138	1	15/08/2014	Archivos argentinos de pediatra	Ceriani Cernadas JM
NCT01305226	0			
NCT00421551	1	24/09/2010	AIDS	Katlama C
NCT01053325	0			
NCT01765452	0			
NCT01882907	1	05/04/2016	Diabetes and metabolisme journal	Ho Kim J
NCT01547598	0			
NCT00544128	1	01/04/2013	Internal Medicine	Nishijama T
NCT01840605	0			
NCT01613326	1	17/01/2014	BMC Pulmonary Medicine	Chapman KR
NCT01896232	1	10/01/2017	JAMA	Block GA
NCT00784017	1	04/05/2018	pediatric blood and cancer	Van der Sluis IM
NCT01474590	1	28/10/2014	british journal of dermatology	Tan J
NCT01763749	1	27/10/2015	Clinical Drug investigation	Lim S
NCT01420549	1	28/07/2020	Current Therapeutic Research	VATTIMO Antonio Carlos
NCT00809146	1	16/02/2012	NEJM	Silbergeit R
NCT01529229	1	13/09/2016	Brazilian journal of othorhinolaryngology	Wandelsen G
NCT02200146	0			
NCT01529541	1	14/01/2015	Diabetes. obesity and metabolism	Jin SM
NCT02458768	1	01/03/2017	Reproductive biomedicine online	Koo HS

NCT01057407	1	27/08/2013	Therapeutic apheresis and dialysis	Akizawa T
NCT01106131	1	07/01/2015	Diabetes. obesity and metabolism	Jin SM
NCT01508325	1	18/08/2016	Hypertension Research	Yang T
NCT00498225	1	01/04/2013	Journal of clinical oncology	Ueno H
NCT01485991	1	05/12/2014	Lancet Infectious disease	Reddy KR
NCT00965094	0			
NCT01378286	1	20/10/2016	Clinical infectious disease	
NCT01961856	1	14/01/2016	Platelets	Alexopoulos D
NCT01120405	0			
NCT00994344	1	21/04/2016	AIDS research and human retroviruses	Santos JR
NCT01505127	1	02/03/2016	BMJ	Murakami K
NCT00440193	1	28/09/2014	Lancet haematology	Prins MH
NCT01342081	0			
NCT00439777	1	28/09/2014	Lancet haematology	Prins MH
NCT00170209	1	02/08/2018	NEJM	Diallo T
NCT01628926	1	12/10/2014	Parkinsonism and related disorders	Mizuno Y
NCT01495702	1	05/06/2014	Lancet Infectious disease	Pozniak A
NCT01431495	0			
NCT01763645	1	01/02/2022	BMC Cancer	Stroyakovskiy DL
NCT01475838	1	05/06/2014	Lancet Infectious disease	Arribas JR
NCT01480284	1	03/04/2017	Hepatolohy research	Koike K
NCT01263015	1	07/11/2013	NEJM	Walmsley SL
NCT00849095	1	04/12/2014	The lancet respiratory medicine	Papi A
NCT01313780	1	11/09/2017	Chinese journal of cancer	Lee KH
NCT00477295	1	08/06/2012	Lancet neurology	Baulac M
NCT01212913	1	07/05/2015	Journal of Diabetes	Jin SM
NCT01768468	1	25/01/2016	Journal of ethnopharmacology	Ha CW
NCT01183104	1	12/04/2017	Diabetes. obesity and metabolism	Terauchi Y
NCT01497366	1	23/04/2013	NEJM	Lawitz E
NCT00809172	1	28/09/2017	The journal of allergy and clinical immunology : in practice	Goujon C
NCT02602028	1	07/04/2016	Arthritis research and therapy	Polat A
NCT00965549	1	18/06/2015	Diabetes. obesity and metabolism	Vora J
NCT00699920	1	01/12/2014	Plos one	Yeka A
NCT01659775	1	11/03/2022	Korean J Intern Med	Der Sheng Sun
NCT01252940	1	28/01/2014	AIDS	Palella FJ Jr

© 2025 Reinaud C et al. *JAMA Network Open.*

NCT01586572	1	17/06/2015	Lancet infectious disease	Mir F
NCT00844337	1	01/04/2015	Lancet global health	Bagui AH
NCT00915382	1	27/07/2015	Annals of oncology	Ryu MH
NCT01228149	1	15/02/2017	Plos one	Lorenz K
NCT01431456	1	18/01/2021	BMJ Open	Lucia van der Veen
NCT01170767	1	02/08/2013	Ophtalmology	Kodijkan L
NCT01294163	1	15/12/2017	Anesthesiology	Hofland J
NCT01382004	1	11/01/2012	The lancet	Mitja O
NCT00784810	0			
NCT00682578	1	21/04/2010	Malaria journal	Awab GR
NCT01084213	1	10/08/2015	Plos one	Tagbor H
NCT00996736	1	13/04/2013	JAMA Opthalmology	Prajna NV
NCT01756339	1	04/02/2016	Lancet Infectious disease	
NCT01181167	1	01/12/2016	Thrombosis journal	Kawai Y
NCT01127360	1	13/09/2014	American academy of ophtalmology	Berg K
NCT02153762	0			
NCT00975143	1	09/05/2015	Osteoporosis international	Hoover KB
NCT01341717	0			
NCT00845728	1	21/08/2013	Lancet respiratory medicine	
NCT01706471	1	01/01/2015	Transplantation	Oh CK
NCT00925587	1	10/02/2014	Nephrology	Roger SD
NCT01170754	1	19/06/2012	Ddigestive diesease and science	McKenna T
NCT00873626	0			
NCT00885079	1	13/03/2013	Ophtalmology	Kinoshita S
NCT01241591	1	05/06/2015	The lancet	Bachelez H
NCT00002651	1	04/04/2013	NEJM	Hussain M
NCT01860560	0			
NCT01113372	1	31/10/2013	JAMA	Feres F
NCT02110238	0			
NCT00764114	1	05/11/2014	Lancet	Bernard L
NCT01428830	1	21/04/2015	Lancet	Barone ma
NCT00774566	1	17/08/2015	Circulation	Luik a
NCT01917591	0			
NCT01323972	1	28/07/2014	Vaccine	Umeh r
NCT01135225	1	15/02/2012	Journal amercican cardiology	Meredith it
NCT01654224	1	17/12/2014	Journal infectious disease	Nace da
NCT00617084	1	16/06/2010	NEJM	Serruys p
NCT02079311	0			
NCT00881959	1	01/12/2014	The Journal of Periodontology	HL Wang

© 2025 Reinaud C et al. *JAMA Network Open.*

NCT00881959	1	01/12/2014	The Journal of Periodontology	HL Wang
NCT01712867	0			
NCT00969436	1	11/09/2015	BMJ open	Lalwani s
NCT01635816	1	18/09/2014	Vaccine	Zaman k
NCT01992094	1	11/07/2016	Human vaccines and immunotherapeutics	Bart s
NCT01867021	0			
NCT01992107	1	14/11/2015	International journal of infectious disease	Hartvickson r
NCT01214889	1	08/07/2016	Journal of korean medical science	Kang jh
NCT01335776	1	06/01/2014	Journal of clinical oncology	Garkand sn
NCT01688921	1	30/05/2014	Lancet	McAllister L
NCT01210261	0			
NCT00992511	0			
NCT00575198	0			
NCT02177032	1	06/06/2018	Plos neglected tropical medicine	Kerdpanich p
NCT01662440	1	18/05/2015	Travel medicine and infectious disease	Jelinek t
NCT02692859	0			
NCT02150915	1	02/05/2018	Acupuncture in medicine	Morais n
NCT03353376	1	28/05/2018	Journal of diabetes research	Singer j
NCT01761175	1	01/01/2016	Anesthesia and anelgesia	Boivin a
NCT02383615	1	15/10/2015	Regional anesthesia and pain medicine	Marian A
NCT01220557	1	09/10/2013	Diabetes research and clinical practice	Hermanns n
NCT00999206	0			
NCT00412516	0			
NCT01126190	1	25/10/2013	Clinical breast cancer	Volovat C
NCT02759003	1	01/12/2017	European Journal of physical and rehabilitation Medicine	Bertella E
NCT00767962	0			
NCT01210040	1	23/11/2017	BMC medicine	Brabin L
NCT01257438	1	08/08/2016	Journal of vascular and interventional radiology	Falk a
NCT00637156	1	17/03/2017	Journal of neurosurgery spine	Gornet mf
NCT01750814	0			
NCT01091246	1	01/07/2012	Pediatric Infectious Disease JournaL	Stan L. Block
NCT02540525	1	15/03/2014	Obstetrics and gynecology	Djehdian Lucyana
NCT02133716	1	19/12/2017	Acta paediatrica	Collados-gomez L

NCT01365494	1	18/02/2015	Human vaccines and immunotherapeutics	Mahendra bj
NCT01951573	0			
NCT01373502	1	25/01/2012	Journal of the american college of cardiology	Carrié d
NCT03410888	0			
NCT01682876	1	15/01/2016	The Pediatric Infectious Disease Journal	William Johnston
NCT00901992	1	28/06/2011	Patient education and counseling	Hermanns N
NCT02467842	1	13/04/2017	Human vaccines and immunotherapeutics	Choi ws
NCT00280475	1	20/06/2018	Journal of Clinical Oncology	Takamasa Kayama
NCT01146743	1	11/01/2012	Gastroenterology	Jang jw
NCT01436396	1	15/10/2016	The Pediatric Infectious Disease Journal	Lopez p
NCT01166711	1	29/07/2015	Journal of interventional cardiology	Zurakowski a
NCT03203408	1	10/12/2019	Plos One	Emmanuel Maheu
NCT01543373	1	13/08/2014	International journal of cardiology	Prati f
NCT01712984	1	19/01/2015	Vaccine	Gorse j
NCT01745549	1	10/12/2014	Current medical research opinion	Valentini m
NCT01010113	1	07/04/2015	Maternal Health. Neonatology. and Perinatology	Lee le y
NCT01700621	1	27/01/2016	Journal of infectious disease	Zaman K
NCT01450449	1	21/09/2015	Journal of clinical oncolgy	Roa w
NCT01944072	1	28/07/2015	Respiratory care	Santos c
NCT01151241	1	01/12/2015	Anesthesiology	Hillen Cruz Eng
NCT02118961	0			
NCT01430611	1	03/12/2020	Human Vaccines & Immunotherapeutics	Feng-Cai Zhu
NCT01753635	1	01/06/2013	Anaesthasia	V. Alexiev
NCT01008891	1	06/04/2015	Foot and ankle international	Daniels TR
NCT02089347	0			
NCT01184079	1	14/12/2013	Vaccine	Lin cj
NCT01696305	1	04/11/2017	International surgery	WY Kim
NCT01475539	1	16/04/2015	Journal of the pediatric infectious diseases society	Li rc
NCT01092507	1	01/06/2014	Pediatric infectious disease	Feroldi e
NCT00931515	0			

NCT01218646	1	12/07/2017	Human vaccines and immunotherapeutics	Greenberg dp
NCT00583375	1	03/07/2013	Journal of Bone & Joint Surgery	Digiovanni w
NCT01411241	1	15/06/2017	The Pediatric Infectious Disease Journal	Flor Irene Rodriguez Melo
NCT01240746	1	01/06/2014	Pediatric infectious disease	Greenberg dp
NCT00616837	1	08/09/2016	BMC health service research	Buvik a
NCT01221337	1	23/06/2016	American journal of kidney diseases	Islam ms
NCT01166685	1	19/11/2014	Circulation	Kaiser c
NCT01661920	1	25/07/2016	JAMA Internal Medicine	Ane Uranga
NCT00833352	1	15/09/2015	European heat journal	Leclercq c
NCT01754558	1	15/09/2017	Acta obsterica gynecologica scandinavica	Rudnicki m
NCT01886807	1	27/07/2016	British journal of anaesthesia	Steiner jw
NCT02097316	0			
NCT01453348	1	07/12/2014	Journal of travel medicine	Alberer m
NCT01209780	1	01/05/2012	Pediatric Infectious Disease Journal	Terry Nolan
NCT01365039	0			
NCT00757107	0			
NCT01443416	1	01/07/2016	Pediatric infectious disease	Truck j
NCT01487213	1	30/10/2014	The Lancet	Oppegaard ks
NCT01129518	1	08/01/2015	Lancet infectious disease	Iro ma
NCT00119067	1	25/12/2013	Vaccine	Wright jg
NCT01346592	1	16/09/2014	Vaccine	Nolan t
NCT02290691	0			
NCT02454114	0			
NCT01130974	0			
NCT01200368	1	01/10/2015	Pediatric infectious disease	Togashi t
NCT01594801	1	17/01/2014	Current medical research opinion	Pfutzner a
NCT01466387	1	09/05/2014	Travel medicine and infectious disease	Alberer m
NCT01162122	1	18/07/2014	Vaccine	Frey se
NCT01144663	1	01/04/2017	Pediatric infectious disease	Merino arribas jm
NCT01066650	1	15/02/2012	Journal of the american college cardiology	Von birgelen c
NCT01000974	1	20/05/2017	Vaccine	Klein np
NCT02214225	1	13/03/2017	Vaccine	Treanor jt

NCT00677235	1	04/07/2016	Journal of vascular and interventional radiology	Haskal zj

© 2025 Reinaud C et al. *JAMA Network Open.*

eTable 2. List of the ClinicalTrials.gov records for the 2022 to 2023 sample

NCT
NCT05577455
NCT05462392
NCT05232825
NCT05686525
NCT05180955
NCT05834855
NCT05608733
NCT05869851
NCT05630755
NCT05496855
NCT05530551
NCT05343546
NCT05850364
NCT05402761
NCT05743764
NCT05890638
NCT05280288
NCT05802888
NCT05622266
NCT05465785
NCT05190172
NCT05774262
NCT05720572
NCT05180864
NCT05686161
NCT05331664
NCT05806346
NCT05206110
NCT05286346
NCT05801146
NCT05610319
NCT05606848
NCT05266066
NCT05398289
NCT05241938
NCT05924438
NCT05234788
NCT05740930
NCT05735431
NCT05758831
NCT05532631

© 2025 Reinaud C et al. *JAMA Network Open.*

NCT05249972
NCT05434962
NCT05780372
NCT05660772
NCT05774288
NCT05863351
NCT05806021
NCT05759065
NCT05859165
NCT05297565
NCT05544565
NCT05519189
NCT05289765
NCT05495048
NCT05610748
NCT05908851
NCT05291585
NCT05794230
NCT05691348
NCT05386030
NCT05527730
NCT05875922
NCT05209412
NCT05439629
NCT05321394
NCT05486793
NCT05631093
NCT05853263
NCT05308420
NCT05819515
NCT05325515
NCT05346068
NCT05304468
NCT05411315
NCT05381350
NCT05736068
NCT05747482
NCT05309993
NCT05412693
NCT05380063
NCT05606315
NCT05447559
NCT05842954
NCT05925309

© 2025 Reinaud C et al. *JAMA Network Open.*

NCT05902793
NCT05604963
NCT05406063
NCT05744193
NCT05329220
NCT05749926
NCT05920005
NCT05205759
NCT05590559
NCT05777759
NCT05655754
NCT05840159
NCT05683054
NCT05249816
NCT05464316
NCT05771116
NCT05178420
NCT05769114
NCT05379114
NCT05773014
NCT05541614
NCT05685043
NCT05290467
NCT05610683
NCT05194137
NCT05175014
NCT05916937
NCT05411237
NCT05550714
NCT05426785
NCT05892887
NCT05273385
NCT05466747
NCT05758987
NCT05210387
NCT05478187
NCT05457933
NCT05627037
NCT05295537
NCT05778214
NCT05490433
NCT05774483
NCT05584007
NCT05904743

© 2025 Reinaud C et al. *JAMA Network Open.*

NCT05387200
NCT05491200
NCT05565222
NCT05805800
NCT05397600
NCT05545787
NCT05765747
NCT05820386
NCT05214807
NCT05454345
NCT05364983
NCT05370183
NCT05820633
NCT05440383
NCT05242133
NCT05590403
NCT05450614
NCT05805917
NCT05506033
NCT05550233
NCT05519956
NCT05400642
NCT05643989
NCT05502809
NCT05309291
NCT05629091
NCT05419609
NCT05254808
NCT05185726
NCT05324852
NCT05645809
NCT05353491
NCT05406908
NCT05865379
NCT05491005
NCT05740605
NCT05841849
NCT05535205
NCT05792449
NCT05757505
NCT05341817
NCT05450705
NCT05440149
NCT05834049

© 2025 Reinaud C et al. *JAMA Network Open.*

NCT05822804
NCT05767606
NCT05840991
NCT05269108
NCT05235139
NCT05273879
NCT05857904
NCT05398679
NCT05771779
NCT05461339
NCT05861791
NCT05425979
NCT05717179
NCT05818579
NCT05642208
NCT05772871
NCT05559671
NCT05670171
NCT05545371
NCT05191771
NCT05212571
NCT05268471
NCT05777850
NCT05761275
NCT05490550
NCT05517850
NCT05365620
NCT05417893
NCT05584020
NCT05377450
NCT05696093
NCT05495217
NCT05686382
NCT05475756
NCT05724056
NCT05420467
NCT05301556
NCT05679856
NCT05721456
NCT05725616
NCT05452616
NCT05755516
NCT05633316
NCT05334095

© 2025 Reinaud C et al. *JAMA Network Open.*

NCT05645198
NCT05394298
NCT05240781
NCT05559840
NCT05534581
NCT05308511
NCT05534399
NCT05187611
NCT05509296
NCT05727111
NCT05461313
NCT05311111
NCT05547529
NCT05262777
NCT05612958
NCT05798702
NCT05806918
NCT05822778
NCT05296161
NCT05861973
NCT05516446
NCT05181046
NCT05608278
NCT05256628
NCT05443984
NCT05516784
NCT05671718
NCT05243602
NCT05902702
NCT05789329
NCT05253677
NCT05804032
NCT05447377
NCT05460377
NCT05476432
NCT05453253
NCT05416853
NCT05329701
NCT05860634
NCT05507229
NCT05546502
NCT05217680
NCT05597540
NCT05809258

© 2025 Reinaud C et al. *JAMA Network Open.*

NCT05799378
NCT05353140
NCT05758740
NCT05728424
NCT05301023
NCT05572736
NCT05243836
NCT05481034
NCT05225636
NCT05590923
NCT05910385
NCT05258851
NCT05643651
NCT05258903
NCT05249192
NCT05587062
NCT05697562
NCT05899010
NCT05242432
NCT05286918
NCT05219318
NCT05776758
NCT05634889
NCT05744232
NCT05247918
NCT05575518
NCT05865457
NCT05762198
NCT05647798
NCT05584228
NCT05844384
NCT05417841
NCT05814341
NCT05242081
NCT05711095
NCT05501496
NCT05702970
NCT05514535
NCT05242835
NCT05766696
NCT05450835
NCT05240469
NCT05423496
NCT05891535

© 2025 Reinaud C et al. *JAMA Network Open.*

NCT05275296
NCT05433701
NCT05574244
NCT05498376
NCT05407870
NCT05898776
NCT05698680
NCT05485480
NCT05764876
NCT05692999
NCT05841836
NCT05804136
NCT05659641
NCT05784428
NCT05767723
NCT05716880
NCT05672901
NCT05263934
NCT05530174
NCT05898581
NCT05851274
NCT05274776

© 2025 Reinaud C et al. *JAMA Network Open.*
