# Supplementary material for: Reporting of Noninferiority Margins on ClinicalTrials.gov: A Systematic Review
Source: JAMA Netw Open. 2025 Apr 7;8(4):e253569. doi: 10.1001/jamanetworkopen.2025.3569 (PMC11976490; doi:10.1001/jamanetworkopen.2025.3569)
Supplement: Supplement 2. — Data Sharing Statement [file jamanetwopen-e253569-s002.pdf]

Data Sharing Statement

Reinaud. Reporting of Noninferiority Margins on ClinicalTrials.gov. *JAMA Netw Open*.
Published April 07, 2025. doi:10.1001/jamanetworkopen.2025.3569

Data
Data available: Yes
Data types: Data (not involving human participants), Data dictionary
How to access data: Corresponding author: Prof. Agnès Dechartres, agnes.dechartres@aphp.fr
When available: With publication

Supporting Documents
Document types: Statistical/analytic code
How to access documents: Corresponding author: Prof. Agnès Dechartres, agnes.dechartres@aphp.fr
When available: With publication

Additional Information
Who can access the data: Any academic researcher requesting the data
Types of analyses: For research purpose
Mechanisms of data availability: After approval of a proposal
